# Severe Effects of Cascara Sagrada

**Published:** 1888-03

**Authors:** R. O. Cotter

**Affiliations:** Macon, Ga.


### SEVERE EFFECT OF CASCARA SAGRADA.

BY R. O. COTTER, M. D., MACON, GA.

I have been very fond of the usual pleasant action of the fluid extract of cascara sagrada, but its very severe and even prostrating action in a couple of cases within the past few months have warned me to be more careful in its administration in future. The first case, a man of 60 years of age, was given a drachm dose at night, after several days' constipation, after an operation for cataract. The dose not acting, he was given the same quantity in the morning following and still again repeated at noon. The bowels then began to purge, and for about 12 hours the action of the drug was so severe as to very closely resemble a regular cholera morbus and greatly prostrated the patient. Of course these doses were perhaps too frequently repeated, and yet I have frequently taken it myself in almost the same way and had no trouble. Within the past few days I prescribed a drachm dose for a lady patient at night, and the one dose was followed by very severe action, with great prostration and feebleness for three or four days. I have always regarded cascara sagrada as a very sure and pleasant cathartic and have never read of any unpleasant consequences following its use, but these cases will make me very careful of it in future.

